# Prospects and challenges of CAR-T cell therapy combined with ICIs

**DOI:** 10.3389/fonc.2024.1368732

**Published:** 2024-03-20

**Authors:** Yufan Lv, Xinyu Luo, Zhuoyi Xie, Jieya Qiu, Jinsai Yang, Yuqi Deng, Rou Long, Guiyang Tang, Chaohui Zhang, Jianhong Zuo

**Affiliations:** ^1^ The Affiliated Nanhua Hospital, Hengyang Medical School, University of South China, Hengyang, China; ^2^ Transformation Research Lab, Hengyang Medical School, University of South China, Hengyang, Hunan, China; ^3^ Computer Institute, Hengyang Medical School, University of South China, Hengyang, Hunan, China; ^4^ The Third Affiliated Hospital, Hengyang Medical School, University of South China, Hengyang, China

**Keywords:** immunotherapy, immune checkpoints, CAR-T cells, combination therapy, solid tumors

## Abstract

Immune checkpoint molecules are a group of molecules expressed on the surface of immune cells that primarily regulate their immune homeostasis. Chimeric antigen receptor (CAR) T cell therapy is an immunotherapeutic technology that realizes tumor-targeted killing by constructing synthetic T cells expressing specific antigens through biotechnology. Currently, CAR-T cell therapy has achieved good efficacy in non-solid tumors, but its treatment of solid tumors has not yielded the desired results. Immune checkpoint inhibitors (ICIs) combined with CAR-T cell therapy is a novel combination therapy with high expectations to defeat solid tumors. This review addresses the challenges and expectations of this combination therapy in the treatment of solid tumors.

## 1 Introduction

Immune checkpoints are a group of signaling pathway molecules which have the ability to regulate the persistence of the immune response (1). The common immune checkpoints include CTLA-4, LAG-3 and PD-1, all of which are widely distributed in solid tumors and have a critical function in the tumor microenvironment (TME) (2–4). Tumor cells could inhibit anti-tumor immune responses by activating the immune checkpoint pathways, but ICIs can block the activation of this pathway, which leads to the enhancement of the function of CAR-T cells, activating this immune response to promote tumor cell clearance (5). In recent years, ICIs have been developed rapidly and marketed one after another, and have been approved in a number of solid tumors such as hepatocellular carcinoma, lung cancer, melanoma, and colon cancer (6). Although ICIs have achieved positive clinical efficacy, some patients do not experience complete remission. For example, Chromosomal instability (CIN) is an important factor affecting the efficacy of ICIs in cancer of unknown primary (CUP). CIN can increase the invasiveness of tumors, leading to a decrease in the therapeutic effect of ICIs. Therefore, more clinical genetic tests are needed to improve the efficacy of ICIS for patients with ICIs resistance (7).

**Figure 1:**
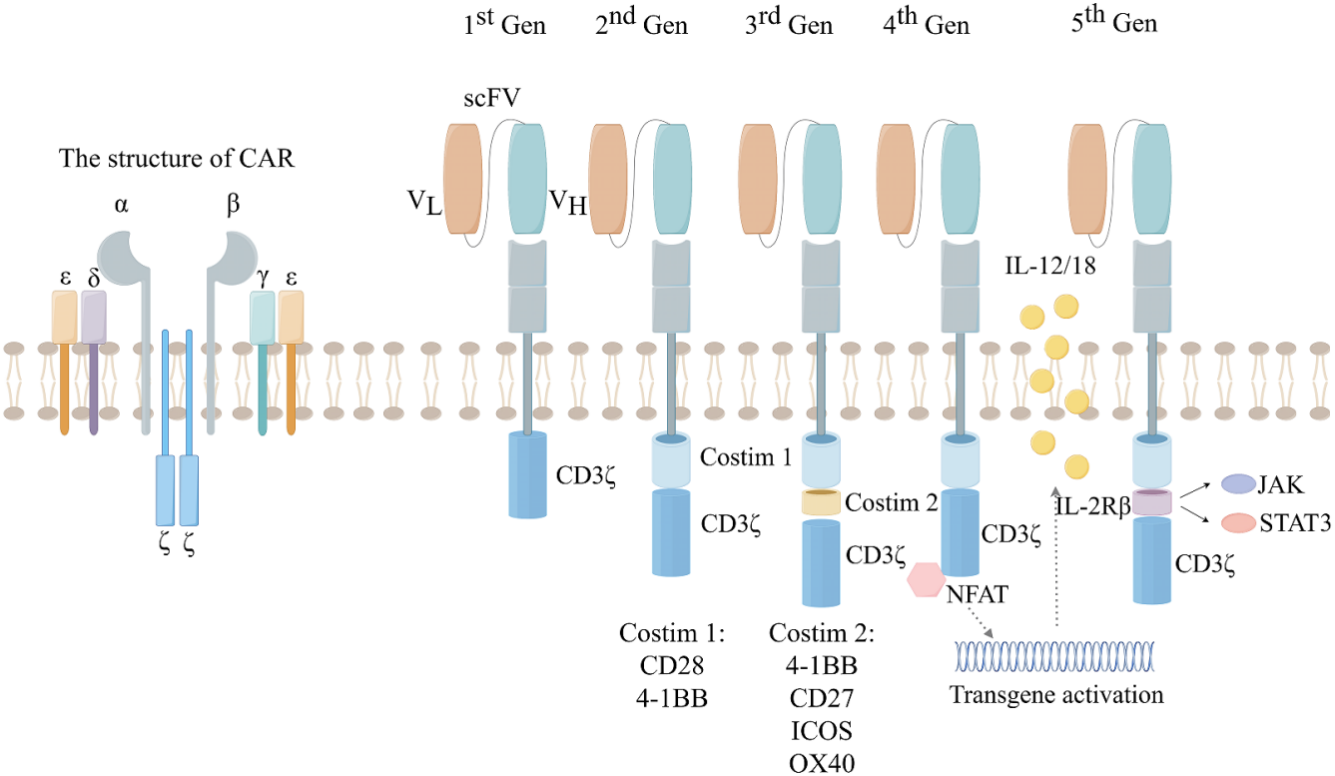
The development of the five generations of CAR. 1^st^:ITAM area containing only CD3. 2^nd^:On the basis of the first generation, a co-stimulatory region (CD28 or 4-1BB) was introduced. 3^rd^: On the basis of the second generation, two co-stimulatory regions (CD28 and 4-1BB) were introduced. 4^th^:Introducing the regulation of CAR-T cell structure, such as cytokines and chemokine receptors. 5^th^: Creating allogeneic CAR-T cells by knocking out endogenous T cell receptors (TCRs) and leukocyte antigen class I molecules (HLAs).

Although CAR-T cell therapy and ICIs belong to the same category of immunotherapy, their mechanisms for treating tumors are completely different. CAR refers to the construction of a specific chimeric antigen receptor containing a single-chain variable fragment (ScFc) that recognizes tumor antigens through bioengineering technology, the researchers then introduced the artificially constructed CAR into the T cells to complete the construction of CAR-T cells (8). Artificially constructed CAR-T cells can precisely target tumor cells, and the CAR-T cells also release various tumor suppressor cytokines, such as IL-2, IL-12, and IL-18, to precisely and efficiently treat tumors (9). As a result, specific receptors loaded on CAR-T cells can bind to target antigens independently of MHC receptors, resulting in strong T-cell activation and powerful anti-tumor responses (10). Because of this, CAR-T cell therapy has achieved excellent efficacy in hematologic tumors, but has been disappointing in solid tumors. The reasons for CAR-T therapy not achieving the desired therapeutic effect in solid tumors are complex. On the one hand, solid tumor cells antigen heterogeneity and the lack of tumor-specific antigen (TSA) make it difficult for CAR-T to target tumor cells. On the other hand, the TME of solid tumors, including abnormal vascular structure and stromal composition, make it difficult for CAR-T to accurately target and kill tumor cells. So far, the development of CAR has gone through five generations (**Figure 1**). However, CAR-T cell therapy still has some limitations, such as antigen escape, off-target phenomenon and lymphokine release syndrome (11, 12). The low cure rate and relatively high side effects in the treatment of solid tumors have become obstacles to the further application of CAR-T.

However, CAR-T cell therapy in combination with ICIs can be an effective strategy to resolve these limitations (13). ICIs can activate the anti-tumor immune function of CAR-T cells, leading to a decrease in the secretion of inhibitory cytokine, and also partially rescues the inhibitory effect of the tumor microenvironment (TME) (14). At the same time, ICIs also activates the function of tumor-infiltrating lymphocytes (TILs) cells and enhances their anti-tumor capacity (15, 16). Combination therapy has been shown to be more efficacious than each drug alone in preclinical studies, supporting its extension to clinical studies. In this review, we summarize the current state of research centered on immune checkpoint inhibitors in combination with CAR-T therapy.

## 2 Issues and challenges of CAR-T in solid tumors

### 2.1 Lack of tumor-specific antigens (antigenic heterogeneity)

Currently, CAR-T cell therapy has made a revolutionary breakthrough in hematologic malignancies due to the specificity of targets for hematologic malignancies (17, 18). Six CAR-T Cell Therapy drugs approved for marketing by FDA, including four that targeting CD19 and two that targeting BCMA (**Table 1**) (19, 20, 22–25). TSAs are antigens that are specific to tumor cells and are expressed only in tumor cells and not in any normal cells. Tumor-associated antigens (TAAs) are antigenic components that are not specific to tumor cells. TAAs can be present in small amounts in normal cells, but can be abnormally expressed in tumorigenic organisms. Hematological tumors have TSA such as CD19 and CD20, while solid tumors have high heterogeneity, making it difficult to obtain ideal TSA for CAR-T cell therapy (26). Thus, the targets of CAR in solid tumors are diverse, such as GPC3 in hepatocellular carcinoma, HER-2 in glioblastoma, CLDN18.2 in pancreatic cancer, and PSMA in prostate cancer (27–29). Therefore, CAR-T cell therapy for solid tumors could only be designed to target such non-specific TAAs. Because of the lack of TSA, CAR-T cell therapy faces the dilemma of “off-target effects” by attacking normal cells (30). For example, HER-2 is highly expressed on the surface of colorectal cancer cells, but is also expressed to a small extent in normal cardiopulmonary tissues. A patient with colon cancer developed acute respiratory distress only 15 minutes after receiving a higher-dose infusion of HER-2 CAR-T cells and died 5 days after treatment (31).

**Table 1:** FDA approved CAR-T cell therapy products.

CAR-T Name	Target	Indications	Time to market	Reference
Kymriah	CD19	BCR-AL/rr DLBCL	2017.08.30	Maude Slet al. (19)
Yescarta	CD19	rr DLBCL/rr FL	2017.10.18	Locke FL et al. (20)
Tecartus	CD19	rr MCL	2020.07.24	Wang M et al. (21)
Breyanzi	CD19	rr DLBCL	2021.02.05	Abramson JS et al. (22)
Abecma	BCMA	rr MM	2021.03.26	Berdeja JG et al. (23)
Carvykti	BCMA	rr MM	2022.02.28	Munshi NC et al. (24)

Furthermore, the killing capacity of CAR-T cells is not only related to the number of tumor cells expressing the target antigen in solid tumors, it is also related to the intensity of tumor surface antigen expression (32). On the one hand, in solid tumors, the expression intensity of target antigens of CAR-T is inherently much lower than that of hematologic malignancies. On the other hand, in solid tumors, infusion of specifically targeted CAR-T cells for treatment may lead to down-regulation of target antigen expression, which prevents CAR-T cells from recognizing tumor cells, thus leading to therapeutic failure (33). The results of the clinical experimental study by Fry et al. also suggest that relapse after treatment with infusion of CAR-T cells targeting CD22 in patients with B-ALL is associated with a decrease in the density of the CD22 locus (34).

### 2.2 Low efficiency of tumor infiltration (physical barrier)

In hematologic malignancies, CAR-T cells can directly contact tumor cells in blood vessels, thereby exerting their effects (32). However, this is not the case in solid tumors, the CAR-T cells need to overcome multiple barriers to bind to surface antigens of solid tumor cells. The CAR-T cells need to cross this physical barrier in multiple steps, each of which is interconnected and tightly coupled. The whole process includes rolling, adhesion, wandering (amoebic movement), and chemotaxis. And it also requires the assistance role of cell adhesion molecules (CAM) and chemokines (35, 36).

In addition to the multiple barriers that CAR-T cells themselves need to traverse. Solid tumor cells also resist CAR-T cell entry in multiple ways. Some of these mechanisms include:1. Abnormal expression of adhesion molecules in the vasculature system reduces CAR-T cell attachment and migration (37–39). 2. Down-regulates the expression of various chemokines (CCL5, CXCL9 and CXCL10). CAR-T cells are dependent on the transport of these chemokines to tumor tissues (40, 41). 3. Dense tumor extracellular matrix prevents CAR-T cells from entering.

Stromal cells such as fibroblasts and macrophages play a key role in the formation of dense extra-tumor stroma. Methylmalonic acid (MMA) secreted by tumor cells further promotes the proliferation of fibrous or connective tissues, while proliferating stromal cells in turn secrete more collagen, proteoglycans, fibronectin, laminin, and so on. These substances form a non-cellular three-dimensional macromolecular network, the tumor-associated extracellular matrix (ECM), which prevents the infiltration of CAR-T cells (42). For example, significantly increased hyaluronic acid (hyaluronic acid) content in TME of pancreatic cancer, which directly affects the pathological type and biological behavior of pancreatic cancer (43–45). Furthermore, fibroblasts secrete fibroblast activation protein (FAP), which can form dense mesenchymal cell tissues to prevent CAR-T cells from entering. ELLEN Pure et al.’s study confirmed that removing the dense mesenchyme of tumors can allow CAR-T cells to directly enter the core of solid tumors. The solid tumors in mouse animal models rapidly regressed and CAR-T cells showed enhanced anti-tumor immune effects (46).

### 2.3 Suppression of the tumor immune microenvironment (immune barrier)

The tumor immune microenvironment (TIME) is a complex and dynamic ecosystem, which is closely related to tumor development, progression and metastasis. The TIME is filled with various immune cells and cytokines. In order to recognize and resolve tumor cells, CAR-T cells must continuously infiltrate the tumor tissue in order in order to achieve antigen-specific binding on the tumor cell surface, thereby exerting a killing effect on tumor cells. While the normal immune microenvironment is balanced between promoters and suppressors, a large number of immunosuppressive cells and cytokines are present in the TIME.

Previous studies have demonstrated that CAR-T cell metabolism is strongly inhibited in the TIME (47–49). The formation of tumor microenvironment mainly originates from the abnormal activation of oncogenes in tumors, resulting in the abnormal expansion of suppressive immune cells and the massive secretion of immunosuppressive factors. Among the cells that make up the immune microenvironment, tumor associated macrophages (TAMs) is a relatively abundant cell subpopulation with a highly plastic phenotype and function. For example, the mutation of P53 can lead to the differentiation of M1-type TAMs to M2-type, and K-Ras(12D) in melanoma can induce the proliferation of CD11 myeloid cells with immunosuppressive function. These T regulatory (Treg) cells and TAMs cells also release reactive oxygen species (ROS), which significantly inhibit the killing response of NK cells and T cells (50). At the same time, fibroblasts secrete higher amounts of metalloproteinases, leading to the shedding of ligands attached to T cells and NK cells (18). All these confirm that immunosuppressive cells severely affect the killing effect of T cells and NK cells. In addition to tumor immunosuppressive cells, suppressor cytokines also play an extremely important role (51). For example, mutations in the BRAF600E gene in melanoma can reduce the production of chemokines CCL3 and CCL4, thereby reducing the ability of tumor killing cells, M2-type TAMs mediate immune escape by secreting TNF-α and IL-10 in order to promote PD-L1 expression, thereby suppressing anti-tumor T cell function. Many other studies have shown that Tregs in tumors inhibit anti-tumor immune responses by suppressing the production of inhibitory cytokines (e.g., IL-10, TGF-β, IL-35) and suppressing anti-tumor immune responses (52, 53).

A number of studies have similarly confirmed this view, Miriam Merad et al. produced CAR-T cells targeting TAMs, destroyed immunosuppressive cells to enhance CAR-T cell function, significantly prolonged progression of solid tumors and enhanced tumor immunity in animal experiments (54). In addition, there are also CAR-T cells made that target Treg cells to disrupt the over-activation of regulatory T cells, and as the Treg cells are reduced, the tumors in the mice are progressively reduced (55). Jiali Yu at al. showed that radio therapy (RT) could reduce the number of hepatic myeloid cells, thereby increasing the infiltration of hepatic T cells, promoting the release of cytokines such as Ki67^+^, interferon-γ (IFN-γ)^+^, and significantly improving the immune microenvironment (56). Similarly, Smith EL et al. have also demonstrated that RT destroys immunosuppressive cells such as Tregs, CAFs, thereby synergizing with CAR-T cells and reducing T cells exhaustion (57). In summary, suppressive immune cells and immunosuppressive factors are closely linked and interact with each other, together constituting the immunosuppressive microenvironment (58).

## 3 CAR-T cells exhaustion

### 3.1 CAR-T cells exhaustion mechanisms

CAR-T cells in the TME are chronically exposed to antigens and gradually enter a state of malfunction, which is called CAR-T exhaustion (59, 60). Many studies have already shown that CAR-T cells exhaustion is involved in a range of tumor development processes, such as drug resistance of tumor immunotherapy, tumor recurrence and metastasis (34). CAR-T cells exhaustion is mainly characterized by loss of effector functions, such as reduced release of cytokines IFN-γ and TNF-α, and loss of proliferative capacity (61). It has been shown that CD8^+^ T cells in malignant tumors all have significant enrichment of genes associated with TCR signaling (Bat-f, Egr2, Ezh2, Irf4, Nfatc1, Nfatc2, Nr4a1, Nr4a2 and Nr4a3) (62–66), confirming the involvement of persistent antigenic stimulation in leading to high PD-1/PD-L1 expression, which directly contributes to CAR-T cells exhaustion. Kelly Kersten et al. showed that CD8^+^ T cells depletion is directly correlated with macrophage abundance in the TME, revealing in detail the spatiotemporal co-evolution of CD8^+^ T cells lymphocytes and macrophages in the immune microenvironment (67).

Currently, researchers have demonstrated that CD38 is a signature molecule for CAR-T cells exhuastion (68, 69). They found that inhibition of CD38 resulted in a significant increase in intracellular NAD^+^ levels, which in turn led to an increase in SIRT expression and acetylase activity. Activation of the CAD38-NAD^+^-SIRT1 pathway resulted in a decrease in HIF-1 stability, thereby inhibiting glucose metabolism in CAR-T cells. In addition, the use of small molecule inhibitors targeting CD38 significantly enhanced the persistence of CAR-T cells (70). Similarly, a large number of tumor cell metabolites in the TME are also involved in this process. For example, tumor cells in AML patients can release large amounts of kynurenine, which can significantly inhibit CAR-T cell activity (71). In addition, many tumor metabolites (mitochondrial ROS, adenosine) contribute to CAR-T cell exhaustion (72, 73). In conclusion, metabolic disturbances in the TME play an important role in the CAR-T depletion process.

CAR-T cells exhaustion does not occur suddenly, and involves a complex process of differentiation. The transition of effector T cells to an exhausted state is accompanied by significant epigenetic reorganization and distinct transcriptional features. T-cell fctor-1(TCF-1), a central transcription factor in early CAR-T cells exhaustion (74, 75). The study shows that Chromatin peaks containing TCF family transcription factor motifs shut down during the transition from a plastic to a dysfunctional stationary state, with a corresponding decrease in TCF-1. Apart from this, there are many common features observed in T-cell exhaustion, where CAR-T cells are functionally exhausted concurrently with and often characterized by high surface expression of inhibitory receptors (CTLA-4, PD-1, TIM-3, LAG-3, and 2B4) on CAR-T cells (61, 76).

### 3.2 Association of ICIs with CAR-T cells exhaustion

During the preparation process of CAR-T cells, it was found that the expression level of PD-1 on the patient’s T cells affects the killing function of the prepared CAR-T cells. Additionally, it was found that PD-1 were highly expressed on many exhausted CAR-T cell epitopes (77). Yamaguchi et al. found that M2-type TAMs in TME induced CAR-T to overexpress PD-1, thereby affecting the PD-1/PD-L1 pathway to reduce the activity of CAR-T cells. Besides, the use of atezolizumab (a PD-L1 inhibitor) led to M2-type TAMs apoptosis, which improved the anti-tumor activity of CAR-T cells (78). Therefore, ICIs not only block the PD-1/PD-L1 axis to enhance CAR-T cell function, but also improve the immune microenvironment and reduce CAR-T cell exhaustion (79–81).

**Figure 2:**
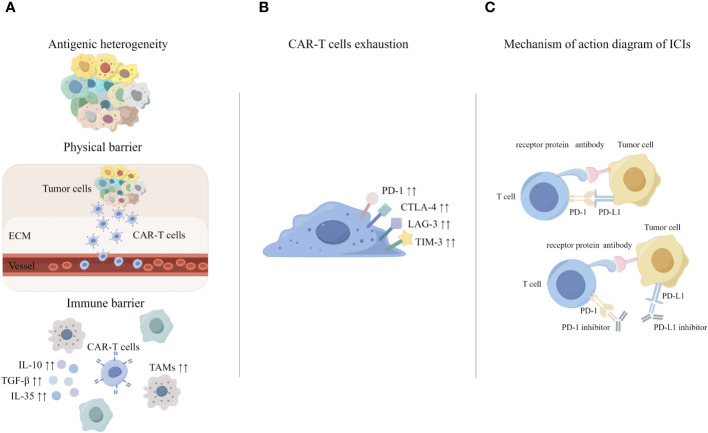
The principle of CAR-T cell therapy combined with ICIs. **(A)** Challenges of CAR-T in solid tumors. **(B)** Mechanisms of CAR-T exhaustion. **(C)** Mechanisms underlying enhanced antitumor activity of PD-1 disrupted CAR-T cells.

Early animal experiments showed that PD-1 inhibitor combined with CAR-T resulted in a significant decrease in the percentage of myeloid derived suppressor cells (MDSC) in the tumor microenvironment of mice (82). In a phase I clinical trial for the treatment of malignant pleural mesothelioma, researchers injected pembrolizumab (a PD-1 inhibitor) after an infusion of CAR-T targeting mesothelin. After 12 weeks of pembrolizumab treatment, the researchers were able to detect CAR-T cells in the patients’ peripheral blood, and the rate of exhausted CAR-T cells was significantly lower (83). Kersten K et al. found that single-target CAR-T therapies gradually produce off-target effects, so CAR-T cell therapy needs to be combined with other immunotherapies (57). In clinical care, patients with relapsed and refractory diffuse large B-cell lymphoma (DLBCL) have disease progression after CAR-T therapy, and patients can achieve remission after receiving additional PD-1 inhibitor therapy (84). Meanwhile, PD-1 inhibitor combined with CAR-T has also achieved impressive therapeutic effects in phase I clinical trials for solid tumors (83). Therefore, a variety of experiments have demonstrated that the PD-1/PD-L1 pathway may play a key role in CAR-T therapy. It was suggested that CAR-T cell therapy combined with ICIs may be a more effective treatment for solid tumors (**Figure 2**).

## 4 Strategies for the clinical application of CAR-T cell therapy in combination with ICIs

### 4.1 Exogenous ICIs combined with CAR-T cell therapy

The strategy for this type of research is to directly combine exogenous ICIs with CAR-T cell therapy. This treatment model involves administering CAR-T cell therapy alone to the patient, followed by injection of ICIs after 1-2 months of clinical observation (85, 86). Back in 2018, there was a new breakthrough in the treatment of malignant brain tumors with CAR-T combined with PD-1/CTLA-4 antibodies. Researchers used two different treatment methods for invasive brain cancer glioblastoma (GBM): CAR-T cell therapy alone and combination therapy. Encouragingly, CAR-T cell therapy in combination with PD-1/CLTA4 antibody showed much higher anti-cancer efficacy than CAR-T therapy alone (87). In addition to this, oncolytic viruses (Ovs) combined with CAR-T cell therapy has achieved promising efficacy in GBM. For example, oncolytic Herpes Simplex Virus HSV-1 (oHSV-1) can significantly increase the release of T-cells and IFN-γ from GBM, which enhances the therapeutic effect of CAR-T. In addition, wang et al. combined CXCL11-loaded oncolytic virus (oAds-CXCL11) with CAR-T cell therapy and found an increase in T-cell and NK infiltration, as well as a significant decrease in M2-type macrophages in mouse GBM model. In September 2021, in a phase I//IIA clinical trial, CAR-T cell infusion followed by the use of Pembrolizumab (a PD-1 inhibitor) in the treatment of refractory B-cell lymphoma produced better clinical efficacy. Twelve patients were evaluated, one in complete remission (CR) and two in partial remission (PR). The experiments also demonstrated that activation and proliferation of CAR-T cells increased (88). In November 2021, Prasad S Adusumilli Prasad S Adusumilli et al. published the results of a phase I clinical trial. This is a clinical trial of CAR T cell therapy in combination with the pembrolizumab in patients with malignant pleural disease, the clinical trial data showed that patients treated with this combination had a median survival of 23.9 months, with a one-year survival rate of 83%, which was much higher than the 17.7 months, 74% one-year survival rate for single CAR-T cell therapy (83).

In a clinical trial of neuroblastoma, researchers have found that CAR-T cell therapy in combination with ICIs showed better T-cell persistence and anti-tumor efficacy than CAR-T cell therapy in combination with chemotherapeutic agents (89). In addition, the combination of ICS with CAR-T has been clinically tested in various solid tumors such as melanoma, non-small cell lung cancer (NCLC) and ovarian cancer.

The advantage of combination therapy is that it can improve the objective remission rate of advanced patients (90–92). However, there are some limitations to this combined treatment strategy. First, the blocking effect of PD-1 inhibitors is transient and requires repeated administration. Second, PD-1 inhibitor can be captured by TAMs before reaching the surface of CAR-T cells, thereby eliminating their ability to block PD-1/PD-L1 pathway. Third, systemic application of ICIs produces strong systemic side effects. Common adverse reactions in patients treated with PD-1 inhibitors include rash, diarrhea, and thyroid dysfunction. Other more serious immune complications associated with ICIs include cardiac complications, neuromuscular disease, and pneumonia (93–96).

As a result, many patients do not achieve the desired results with CAR-T therapy followed by anti-PD-1 therapy. Moreover, some studies have shown higher relapse rates in patients after using ICIs. What’s more, Minagawa, K et al.’s study showed that the role of PD-1 in lung cancer is exactly the opposite, as blocking PD-1 actually promotes tumor cell proliferation (97, 98). Therefore, due to the ineffectiveness and relatively high number of side effects of this treatment, researchers are actively seeking alternative treatments.

### 4.2 CAR-T therapy with auto endocrine immune checkpoint antibodies

The design idea of this study is to allow CAR-T cells to express the CAR while allowing the T cells to additionally express the PD-1/PD-L1 antibody scFv. In this way the CAR-T cells are allowed to secrete the PD-1/PD-L1 antibody to block the immune cell PD-1 and the tumor cell PD-L1. Compared to the combination of exogenous ICIs and CAR-T cell therapy, this CAR-T cell that can secrete ICIs on its own has two advantages. On the one hand, PD-1 antibodies secreted by CAR-T cells can break through the limitations of the immune microenvironment of solid tumors, reduce the depletion of CAR-T cells, and play a cooperative role in killing tumors. On the other hand, systemic PD-1 monoclonal antibodies in combination with CAR-T can produce various side effects such as immune pneumonitis, cytokine storm, etc (99). However, CAR-T cell therapy with endocrine immune checkpoint antibodies is able to precisely hit tumors and secrete ICIs only at the tumor site, avoiding toxic side effects at other sites.

In October 2018, Rafiq S et al. transformed CAR-T cells to successfully secrete PD-1-blocking single-chain variable fragments (scFv). Researchers construct mouse solid and hematologic tumor models. It was found to increase the anti-tumor activity of CAR-T cells while significantly reducing immunotoxicity compared to the effects of combination therapy with CAR-T cells and ICIs (100).

In February 2021, Qian et al. used autocrine PD-1 antibody combined with CAR-T for the first time in the treatment of advanced refractory ovarian cancer. The results showed that, this CAR-T cells not only possesses the function of killing tumor cells, but also triggers the local immune effect of the tumor, and improves the TIME. The progression-free survival (PFS) and overall survival (OS) of the patients reached 5, 17 months (101). In March 2023. The PD-1 nanobody-targeted mesothelin CAR-T cell injection (BZD1901) formally entered the phase I and II clinic trial.

In August 2023, Chen et al. found that autocrine PD-1 antibody combined with CAR-T cell-targeted delivery of single-chain antibody could enhance the anti-tumor efficacy of colorectal cancer in mice (102).

Therefore, the research of autocrine PD-1 CAR-T cells is unique and has achieved better efficacy in isolated experiments and mouse animal models. However, more clinical trials are still needed to verify its efficacy.

Clinical trials registered in Clinicaltrial.gov and www.Chictr.org.cn, using PD-1 expression on CAR-T cells for cancer treatment are summarized in **Table 2**.

**Table 2:** List of registered clinical trials expression ICIs CAR-T cells/T cells to treat cancer.

NCT number	CAR-T product	Target antigen	Disease	Clinical trial phase	Status	Location
NCT03179007	CTLA-4 and PD-1	MUC-1	Solid Tumor	1	Recruiting	Ningbo China
NCT03182816	CTLA-4 and PD-1	EGFR	NCLC	1	Recruiting	Ningbo China
NCT02862028	PD-1	EGFR	NCLC	1/2	Recruiting	Shanghai China
NCT03615313	PD-1	MSLN	Solid Tumor	1/2	Recruiting	Shanghai China
NCT03030001	PD-1	MSLN	Solid Tumor	1/2	Recruiting	Ningbo China

### 4.3 Immune checkpoint genes disrupted CAR-T cells

#### 4.3.1 PD-1/PD-L1 axis

PD-1/PD-L1 is an important signaling pathway in the tumor immune response (103). Es ther Schoutrop et al. showed that CAR-T cells in ovarian cancer had high expression of PD-1 and LAG-3, while the expression of the corresponding receptor PD-L1 was up-regulated in tumor cells. In addition, it has been shown that folate receptors (FR) are overexpressed in more than 90% of ovarian cancers (104). Consequently, researchers have been developed three generations of anti-αFR CAR constructs; anti-αFR.CD3ζ, anti-αFR.CD28.CD3ζ, and anti-αFR.CD28.4-1BB.CD3ζ. Compared to the other two CAR designs, NK92 cells expressing αFR28BBζ exhibited greater antigen specificity and proliferation, and their antigen-induced apoptosis rate was significantly reduced (105). After blocking the PD-1-L1 axis, more functional CAR-T cells generated (106). Elaine Lau et al. showed that CAR-T cells with PD-1 knocker-out (KO) exhibited a lower depletion phenotype and dysfunction, along with longer survival time *in situ* tumor model mice with B-cell malignancy (107). Zhu et al. showed that CAR-T cells with PD-1 KO exhibited enhanced killing ability in an animal glioma model of the brain (108). In August 2018, Guo et al. found that infusion of PD-1 KO CAR-T cells showed stronger killing ability of hepatocellular carcinoma in animal tumor models (109). Therefore, several studies have proved that blocking the PD-1/PD-L1 axis can effectively enhance the function of CAR-T cells, and clinical trials of CAR-T cell therapy targeting PD-1 have been conducted one after another.

Elaine Lau et al. developed a class of allogeneic CAR-T cells with PD-1 KO (CB-010). CB-010 demonstrates superior performance in the treatment of relapsed/refractory Hodgkin lymphoma. These allogeneic CAR-T cells have the advantage of reduced cost and elevated number of applicable patients, but there is a risk of graft-versus-host disease (GvHD). Therefore, CB-010 was prepared by knocking out the TRAC gene to reduce the risk. In May 2022, results from a Phase I clinical trial showed that after 16 patients with relapsed or refractory lymphoma were treated with CB-010 (PD-1 knockout CAR-T therapy), 11 were in complete remission (CR) and 4 were in partial remission (PR). The overall remission rate (ORR) was 94% (107).

Clinical studies on blocking the PD-1/L1 axis are mainly conducted through PD-1 KO. However, some researchers are against PD-1 KO, arguing that cells cannot expand or grow after complete removal of the PD-1 gene, while CAR-T cells with silence of PD-1 are able to expand well *in vitro* without affecting the anti-tumor properties of CAR-T cells.

Wei et al. showed that PD-1 may play an important role in maintaining the normal proliferation and differentiation of T cells, and PD-1 KO may weaken the anti-tumor function of T cells by inhibiting their proliferative activity (110). R S Kalinin et al. found that PD-1 KO resulted in faster terminal differentiation of CAR-T cells as well as accelerated exhaustion of CAR-T cells, while proliferative viability was significantly decreased compared to PD-1 silence. Moreover, PD-1 KO was significantly accompanied by upregulation of the exhaustion marker TIGIT (111).

In conclusion, the PD-1/PD-L1 pathway plays an important role in CAR-T cell depletion, proliferation, and apoptosis. However, whether to silence or Knock-out PD-1 in the preparation of CAR-T cells is still controversial, more research is still needed.

Clinical trials registered in Clinicaltrial.gov and www.Chictr.org.cn, using PD-1 knockout CAR-T cells for cancer treatment are summarized in **Table 3**.

**Table 3:** List of registered clinical trials using PD-1 gene disturbed CAR-T cells/T cells to treat cancer.

NCT number	CAR-T product	Target antigen	Disease	Clinical trial phase	Status	location
NCT03525782	PD-1KO CAR-T	MUC1	NCLC	1/2	Recruiting	Guangzhou China
NCT05812326	PD-1KO CAR-T	MUC1	Breast Cancer	1/2	Recruiting	Guangzhou China
NCT03706326	PD-1KO CAR-T	MUC1	Esophageal Cancer	1/2	Recruiting	Guangzhou China
NCT03545815	PD-1/TCR KO	MSLN	Solid Tumor	1	Recruiting	Beijing China
NCT05732948	PD-1 Silence CAR-T	PSMA/PSCA	Prostate Cancer	1	Recruiting	Suzhou China
NCT03747965	PD-1 KO CAR-T	MSLN	Solid Tumor	1	Recruiting	Beijing China

#### 4.3.2 Other immune checkpoints

Compared to CAR-T cell therapy without immune checkpoint disturbed, CAR-T cells with LAG-3 blockade did not positively affect CAR-T cells function *in vitro* experiments, and the same experimental results were also presented in animal models (112). Sangya Agarwal et al. showed that the CD28 signaling pathway could be inhibited by knocking-out the CTLA4 gene in CAR-T cells. In addition, it also enhanced the anti-tumor activity and CAR expression of CAR-T cells. CAR-T cells with CTLA4 blockade exhibited higher proliferative capacity and better anti-tumor efficacy compared to CAR-T cells with PD-1 blockade (113).

In addition to this, Julia Carnevale et al. explored a novel immune checkpoint gene (RASA2), which promotes T cells activation and enhances their antigenic sensitivity, as well as their proliferative capacity and effector function in a variety of immunosuppressive settings. Removal of RASA2 prolonged survival in mice with liquid or solid tumors in a variety of preclinical T-cell receptor models and CAR - T cell therapy. RASA2 exhibits superior performance compared to other immune checkpoint blockers (PD-1) (114).

Although the blockade of immune checkpoints such as CTLA4 and RASA2 in CAR-T cells has achieved better results in cells, there is still a lack of clinical trials to validate their efficacy.

#### 4.3.3 Multi-target blockade

Huang RY et al. found that when PD-1 were individually blocked in metastatic ovarian cancer cells, other immune checkpoints (LAG-3, CLTA-4) were upregulated accordingly (115).Because of this negative feedback mechanism of immune checkpoints, some researchers believe that research can be explored in the direction of multi-target blockade (116). Lee YH et al. evaluated CAR-T Cell therapy in the context of four different checkpoint combinations blockade: PD-1/TIM-3, PD-1/LAG-3, PD-1/CTLA-4, and PD-1/TIGIT. The study showed that PD-1/TIGIT down-regulated CAR - T cells were found to have a unique synergistic anti-tumor effect. Importantly, functional experiments and phenotypic analyses indicated that PD-1 down-regulation enhanced short-term effector function, while TIGIT down-regulation was primarily responsible for maintaining the hypo-differentiated state, providing a potential mechanism for the observed synergistic effect (116). There are also studies showing that CAR-T cells with blockade of CTLA-4 and LAG-3 exhibit stronger anti-tumor activity in various experimental animal models. In addition to this, CAR-T cells will express CAR on their surface for a longer period of time, its proliferative capacity will also be enhanced (112, 113). CAR-T cells with multi-target blockade undoubtedly show better prospects. At present, there is still a lack of clinical trials to confirm the effectiveness of CAR-T cell therapy with multi-target blockade.

### 4.4 New delivery systems

*CRISPR-Cas9* is a common method to facilitate precise integration of target se quences (117, 118). The usual CRISPR gene editing system uses guide RNA (guide RNA) to mediate the cleavage of genome-specific loci by Cas enzymes. Previous *CRISPR-Cas9* technologies typically use lentiviral packaging methods to insert CAR sequences randomly into the cellular genome, potentially affecting normal gene expression and increasing the risk of oncogenic insertion mutagenesis. Specific responses to DNA of viral origin often impede CAR expression (119–122), and virus manufacturing is often costly (123).

Li et al. used CRISPR-Cas9-mediated homology directed repair (HDR) to precisely knock out PD-1, and then used HDR technology to insert exogenous CAR into the original PD-1 locus. CAR-T cells constructed by this gene editing method (PD-119BBZ) do not carry the risk of exogenous CAR insertion. Besides, this allows precise insertion without the use of viral vectors. Such CAR-T cells (PD-1 19bbz) showed more powerful and longer-lasting killing power compared with lentivirus-infected CAR-T cells (LV-19BBZ). The complete remission rate in patients with relapsed refractory lymphoma was 87.5%, and the objective remission rate reached 100%, by far the best clinical results in global CAR-T cell therapy for refractory relapsed lymphoma with the highest remission rate and low toxicity (124).

In addition, Jennifer Doudna et al. improved Cas9 specificity using a CRISPR hybrid RNA-DNA (chRDNA). The case is guided to recognize genome-specific loci by a heterogeneous sequence of DNA and RNA sequences spliced together. This RNA-DNA heteroduplex sequence significantly reduces off-target gene editing by the gene editing system. This approach provides new ideas for making CAR-T using chRNAs in the clinic (125). The CAR-T cells injection(CB-010) developed by Elaine Lau et al. were edited using chRDNA. The drug performed well in a Phase I clinical trial in refractory B-cell non-Hodgkin’s lymphoma, with an overall remission rate (ORR) of 94% and a complete remission (CR) rate of 69% in treated patients (107).

Michaael Mitchell et al. developed a lipid nanoparticle platform (LNP) that delivers both CAR-mRNA and siRNA targeting PD-1 to T cells. In this way, they generated CAR-T cells with transient CAR expression and PD-1 interference, without altering the overall activation state of the T cells. This delivery method, which can restore the expression of immune checkpoints, can significantly reduce autoimmune risk (126).

Thus, precise gene editing systems are always advancing, and the improvement of gene editing technology has greatly increased the chances of breakthroughs in solid tumors.

### 4.5 Other novel immune combination therapies

Although CAR-T combined with ICIs has achieved a promising clinical outcome, other new immunologic combination therapy strategies have also achieved surprising clinical results. For example, CAR-T combined with Ovs, CAR-T combined with interleukin, and so on. In 2022 Apr, Richard Gvile et have linked two immunotherapeutic approaches, CAR-T and OVs. The researchers loaded the OVs directly onto CAR-T cells, they found that Ovs can activate the CAR-T through the TCR on the CAR-T, and the CAR-T cells can bring the Ovs to the tumor, thus exerting a synergistic effect to kill the tumor. The experiment showed that the addition of OVs dramatically increased the proliferative capacity of CAR-T in mice, and the number of CAR-T cells in spleen, tumor and blood increased dramatically. In addition, animal studies have shown that this combination of treatments extends the lifespan of mice with intracranial gliomas (127). In 2022 Jul, the results of Lushun Chalise et al. also demonstrated that this treatment can significantly arrest the growth of GBM and significantly improve the survival rate of mice (7). There is no doubt that this combination therapy has achieved impressive efficacy in animal experiments, however, it has not yet been carried out in humans. Its effectiveness still needs to be supported by extensive clinical trial data.

## 5 Conclusion

CAR-T cell therapy has proven to be a highly effective strategy for the treatment of hematologic malignancies. However, this treatment method does not perform well in solid tumors, this is mainly due to the fact that solid tumors are characterized by antigenic heterogeneity and immunosuppressive microenvironment, which can damage the tumor-killing function of CAR-T cells. With all these factors, CAR-T cells will eventually go to exhaustion. Among the various strategies to enhance the function of CAR-T cells, CAR-T cell therapy combined with ICIs is a more promising option. This combined treatment strategy is highly theoretically feasible and has achieved good therapeutic results in both animal and clinical trials. However, it has not yet been applied to clinical treatment on a large scale. Therefore, more advanced CAR-delivery systems (non-viral plasmids, electro transfer, LNP) in combination with multiple immune checkpoints (PD-1, CLTA-4, LAG-3, etc.) disturbed can be used, and then multi-center clinical trials can be conducted. We believe that this treatment strategy will have a transformative impact on the treatment of solid tumors in the future.

## Author contributions

YL: Writing – review & editing. ZX: Writing – review & editing, Funding acquisition. XL: Writing – review & editing, Data curation. JQ: Writing – original draft, Software. RL: Writing – original draft, Investigation. JY: Writing – original draft, Software. YD: Writing – review & editing, Investigation, Funding acquisition. GT: Writing – original draft, Funding acquisition. CZ: Writing – review & editing, Resources. JZ: Writing – original draft.
